# Prevalence of Mediterranean fever gene mutations in clinically suspected FMF patients in Algeria

**DOI:** 10.1186/1546-0096-13-S1-P94

**Published:** 2015-09-28

**Authors:** D Ait-Idir, F Bouldjennet, R Taha, H El-Shanti, B Djerdjouri

**Affiliations:** 1 Université des Sciences et de la technologie Houari Boumedienne, Biologie Moléculaire et cellulaire, Bab-Ezzouar, Algeria; 2 Université M'Hamed Bougara de Boumerdès, Biologie, Boumerdès, Algérie, Algeria; 3 Qatar Biomedical Research Institute, Medical Genetics Center, Doha, Qatar

## Introduction

The familial Mediterranean fever (FMF, OMIM 249100) is an autosomal recessive auto-inflammatory disease primarily occuring in Armenian, Turkish, Jewish and Arabic populations. The first clinical symptoms of FMF usually appear in childhood. The chronic relapsing inflammation of the serous membranes leads to febrile attacks often associated with abdominal, joint and/or chest pains. The most common mutations associated with FMF were identified in exon 10 of *MEFV* located on the short arm of chromosome 16p13.3. *MEFV* consists of 10 exons and encodes for pyrin/marenostrin involved in the regulation of NLRP3-inflammasome activity.

## Objectives

In Algeria, FMF is clinically well diagnosed but the disease-causing variations in *MEFV* remain poorly explored. This study aimed to explore the most recurrent mutations in exon 10 in suspected Algerian FMF patients.

## Patients and methods

This study included 84 unrelated Algerian patients (42 males and 42 females) aged between 2 to 56 years. All the patients were recruited from Algerian hospitals and were clinically suspected to have FMF. Genomic DNA was extracted from peripheral blood samples using a standard protocol. Three mutations p.M694I, p.M694V and p.M680I were initially analyzed by PCR-ARMS. In the 84 patients, resequencing the entire coding region of exon 10 was performed on the amplified products on an ABI Genetic Analyzer for confirmation of the identified mutations and identification of others.

## Results

Genetic analysis showed that 33/84 (39.28%) of patients carried at least one mutation in exon 10. The most recurrent mutation was p.M694I which accounted for 18.45% of the total alleles (n= 31/168), followed by p.M680I (8.33%; 14/168), p.M694V (2.38%; 4/168) and p.A744S (2.38%; 4/168). The allele p.M694I accounted for 58.49% of the mutant alleles. Among the patients with mutations, 9 patients were homozygous, of them 7 were p.M694I/p.M694I, 11 were compound heterozygous, of them 9 were p.M694I/p.M680I and 13 were heterozygous. In the rest of the patients, no mutation could be identified.

## Conclusion

The current study shows clearly the predominance of the p.M694I mutation among the Algerian FMF patients which confirms our precedent results. The mutational profile identified here offers a tool for guiding the molecular diagnosis of FMF in Algeria.

